# An Eye on the Wnt Inhibitory Factor Wif1

**DOI:** 10.3389/fcell.2018.00167

**Published:** 2018-12-06

**Authors:** Lucia Poggi, Simona Casarosa, Matthias Carl

**Affiliations:** ^1^Laboratory of Molecular and Cellular Ophthalmology, Centre for Integrative Biology, University of Trento, Trento, Italy; ^2^Laboratory of Neural Development and Regeneration, Centre for Integrative Biology, University of Trento, Trento, Italy; ^3^Laboratory of Translational Neurogenetics, Centre for Integrative Biology, University of Trento, Trento, Italy

**Keywords:** Wif1, Wnt signaling, zebrafish, eye, retina, cancer

## Abstract

The coordinated interplay between extrinsic activating and repressing cell signaling molecules is pivotal for embryonic development and subsequent tissue homeostasis. This is well exemplified by studies on the evolutionarily conserved Wnt signaling pathways. Tight temporal and spatial regulation of Wnt signaling activity is required throughout lifetime, from maternal stages before gastrulation until and throughout adulthood. Outside cells, the action of numerous Wnt ligands is counteracted and fine-tuned by only a handful of well characterized secreted inhibitors, such as for instance Dickkopf, secreted Frizzled Related Proteins and Cerberus. Here, we give an overview of our current understanding of another secreted Wnt signaling antagonist, the Wnt inhibitory factor Wif1. Wif1 can directly interact with various Wnt ligands and inhibits their binding to membrane bound receptors. Epigenetic promoter methylation of Wif1, leading to silencing of its transcription and concomitant up-regulation of Wnt signaling, is a common feature during cancer progression. Furthermore, an increasing number of reports describe Wif1 involvement in regulating processes during embryonic development, which so far has not received as much attention. We will summarize our knowledge on Wif1 function and its mode of action with a particular focus on the zebrafish (*Danio rerio*). In addition, we highlight the potential of Wif1 research to understand and possibly influence mechanisms underlying eye diseases and regeneration.

## Introduction

**FIGURE 1:**
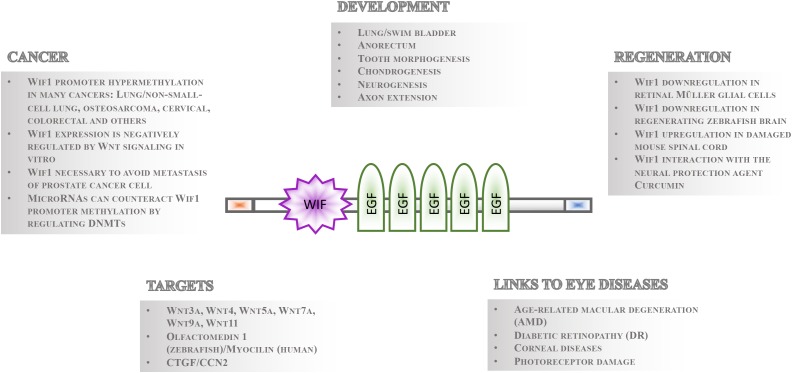
Overview of Wif1 structure and function. The 379 aa Wif1 protein comprises an *N*-terminal signal sequence (orange box), a 150 aa Wif-domain, five epidermal growth factor (EGF)-like repeats, and a 45 aa long hydrophilic tail (blue box) at the *C*-terminus (Hsieh et al., 1999). Wif1 interacts with a number of proteins and has been linked to various processes in embryonic development, regeneration, cancer, and eye diseases as indicated.

Gene regulatory mechanisms facilitate the fundamental necessity that not all cells express and activate all genes and signaling pathways at the same time. Even in the presence of pathway components within a cell, the temporal control of signaling activity needs to be tightly controlled during development and tissue homeostasis. This can occur outside the cells such that ligands are prevented to bind receptors by secreted inhibitory molecules. These repressor proteins can act by directly binding to the ligand or by interacting with their receptor. Studies of the Wnt signaling cascades have been particularly informative as to the mechanisms of extrinsic control of signal activation by either of the nineteen Wnt ligands in mammals and twenty-six in teleosts (Kawano and Kypta, 2003; Beretta et al., 2011; Cruciat and Niehrs, 2013). The secreted repressors interacting with Wnt ligand receptors that have been identified so far include Dickkopf (Dkk) (Glinka et al., 1998; Niehrs, 2006), Sclerostin (Sost)/Wise (Sostdc1) (Itasaki et al., 2003; Li et al., 2005; Semenov et al., 2005) and Insulin growth factor binding protein 4 (Igfbp4) (Zhu et al., 2008). Conversely, those that directly bind to Wnt ligands comprise secreted Frizzled Related Proteins (sFRPs) (Hoang et al., 1996), Cerberus (Bouwmeester et al., 1996) and Wnt inhibitory factor 1 (Wif1) (Hsieh et al., 1999). Among these, Wif1 has perhaps received the least recognition. Its function is, however, implicated in various crucial processes during eye development and homeostasis (Hunter et al., 2004; Park et al., 2014), neurogenesis and axon extension (Hunter et al., 2004; Nakaya et al., 2008), lung and anorectal development (Xu et al., 2011; Ng et al., 2014), tooth morphogenesis (Lee et al., 2015), chondrogenesis (Surmann-Schmitt et al., 2009), stem cell maintenance (Ding et al., 2008; Nakatsu et al., 2011), regeneration and cancer (Wissmann et al., 2003; Lim et al., 2017; Figure 1). The Wif1 protein structure exhibits intriguing features suggesting that its full spectrum of action and importance has yet to be uncovered. Its expression is largely conserved from teleosts to human. This is a prerequisite for using genetically amenable animal models such as the zebrafish for studying Wif1 function *in vivo* to better understand the pathophysiology of Wif1 linked human diseases needed for therapy development.

## Wif1 Characteristics

Wif1 was identified as an expressed sequence tag in the human retina and was functionally described as a negative regulator of canonical or Wnt/beta-catenin signaling in 1999 (Hsieh et al., 1999). However, Wif1 is able to physically interact with both canonical and non-canonical Wnt ligands such as Drosophila wingless, and vertebrate Wnt3a, Wnt4, Wnt5a, Wnt7a, Wnt9a, and Wnt11 (Hsieh et al., 1999; Surmann-Schmitt et al., 2009). In addition, Wif1 binding to zebrafish glycoprotein Olfactomedin1 (Nakaya et al., 2008) and to connective tissue growth factor (CTGF/CCN2) was reported *in vitro* (Surmann-Schmitt et al., 2012). The 379 amino acids (aa) Wif1 protein comprises an *N*-terminal signal sequence, a unique and conserved 150 aa Wif-domain, five epidermal growth factor (EGF)-like repeats, and a 45 aa long hydrophilic tail (Hsieh et al., 1999; Figure 1). Crystal structure analysis of the Wnt ligand binding *N*-terminal part of the Wif1 domain (Wif1WD) and its interaction with Wnt3a revealed that the EGF domains of Wif1 are required for Wnt ligand binding (Malinauskas et al., 2011). In addition, EGF domains II–V have heparan sulfate proteoglycan (HSPG) binding properties. HSPGs strengthen the interaction between Wif1 and Wnt ligands (Avanesov et al., 2012), but they are also known to modulate diffusion of morphogens with which they interact such as Wnt proteins (Panakova et al., 2005; Yan and Lin, 2009). Thus, in addition to Wnt ligand binding, Wif1 might well influence the generation of morphogen gradients and/or protect cells within such gradients from signal cascade activation. This feature might not be restricted to Wnt ligands. The Drosophila Wif1 ortholog “Shifted” promotes hedgehog signaling (Glise et al., 2005; Gorfinkiel et al., 2005), and this was hypothesized to potentially occur also in vertebrates (Avanesov et al., 2012).

Intriguingly, a pocket for phospholipids was identified in the Wif1WD domain (Malinauskas et al., 2011) binding 1,2-dipalmitoyl-phosphatidylcholine (DPPC). This suggested that Wif1 shares similarities with lipoprotein particles that can sequester Wnt3a-linked lipids (Willert et al., 2003; Panakova et al., 2005; Malinauskas et al., 2011). However, since the Wif1WD pocket already binds DPPC, the exchange with a Wnt ligand-bound palmitoleoyl moiety would require energy in the aqueous environment and would therefore rather be unlikely. It was hypothesized that the lipid containing pocket in the Wif-domain may provide conformational flexibility and thereby indirectly contribute to expose the appropriate surface for ligand binding (Malinauskas et al., 2011). Interestingly, also RYK-receptors involved in non-canonical Wnt signaling contain Wif-domains, raising the possibility that the lipid pocket could also be used directly for the binding of moieties attached to Wnt ligands (Kawano and Kypta, 2003; Malinauskas and Jones, 2014).

## Wif1 Epigenetics and Cancer

The Wnt pathway is well known for regulating cell stemness in many organs and tissues including bone, intestine and skin (Steinhart and Angers, 2018). In these and several other tissues, deregulation of various Wnt pathway components has been implicated in cancer occurrence and/or recurrence (Tai et al., 2015; Nusse and Clevers, 2017). One of these components is Wif1, which is downregulated in prostate, breast, lung and bladder cancers, as shown by RNA microarray analysis (Wissmann et al., 2003). Genomic studies identified a CpG island in the human Wif1 promoter 1,5 kb upstream of the Wif1 gene (Reguart et al., 2004). Methylation of CpG islands is one of the major modes of inactivating tumor suppressor genes in cancer (Herman and Baylin, 2003). Indeed, hypermethylation of the Wif1 promoter, leading to Wif1 silencing (and thus activation of Wnt/beta-catenin signaling), was shown to be associated with various types of cancers such as lung cancer, in particular non-small cell lung cancer (NSCLC) (Wissmann et al., 2003; Tan et al., 2013; Zheng et al., 2016; Guo et al., 2017), osteosarcoma formation (Kansara et al., 2009), colorectal cancer (Hu et al., 2018), cervical cancer (Ramachandran et al., 2012) and others (Figure 1). In NSCLC, Wif1 promoter hypermethylation can be counteracted by microRNAs, which negatively regulate DNA methyltransferases in a regulatory feedback loop (Tan et al., 2013). The epigenetic silencing of secreted Wnt pathway inhibitors related to cancer appears not restricted to Wif1 but holds true also for most other members such as Dkk1-3, Sost, Igfbp4 or sFRP1-5 (Mazieres et al., 2004; Aguilera et al., 2006; Roman-Gomez et al., 2007; Sato et al., 2007; Elston et al., 2008; Kongkham et al., 2010; Fellenberg et al., 2013; Gopal et al., 2013). In addition, Wif1 function is required to prevent metastasis of cancer cells. Prostate cancer (PCa) cells can invade bone tissue. In PCa cell lines, *Wif1* expression is spontaneously downregulated by promoter hypermethylation. Restoring *Wif1* expression, however, leads to a reduction in cell invasiveness and motility by upregulation of epithelial markers (Yee et al., 2010). These studies are encouraging for the development of cancer therapies. For instance, targeted disruption or addition of CpG islands in the Wif1 promoter using genome editing techniques would be informative with respect to the resulting cell behaviors in established Wnt cancer models. In parallel, effects at the developmental level can be analyzed *in vivo* in the physiological environment of the zebrafish. Such complementing studies in zebrafish would not only be interesting regarding the epigenetic regulation of genes in general, but would at the same time give important insights into potential side-effects when developing therapies.

Expression profiling experiments have revealed that Wif1 is a downstream target of Wnt/beta-catenin signaling suggesting that Wif1 may act as a feedback inhibitor (Wissmann et al., 2003; Reguart et al., 2004; Vaes et al., 2005; Boerboom et al., 2006; Zirn et al., 2006; Kansara et al., 2009). Thus, Wif1 could be a central player in the dynamic control of Wnt signaling through a regulatory feedback mechanism. Wif1 also plays roles during embryonic development and some evidences collected mainly in mice and zebrafish implicate that Wif1 is similarly self-regulating its own expression during developmental processes (Diep et al., 2004; Yin et al., 2012). Such Wif1 regulatory feedback loops can involve hedgehog (Hh) such that Hh positively regulates *Wif1* expression to inhibit Wnt signaling. In turn, Wnt signaling maintains *Hh* expression. This mechanism is important for swim bladder development in the zebrafish (Yin et al., 2012; Figure 1). Wif1 morpholino knockdown reduces cell proliferation resulting in defective swim bladder development such that epithelium and mesenchyme growth are inhibited, smooth muscle differentiation is abolished and the organization of mesothelium is perturbed.

## *Wif1* Expression and Function in Embryonic Development

Zebrafish *Wif1* starts to be expressed in the presumptive paraxial mesoderm during late gastrulation (Thisse and Thisse, 2005). During subsequent neurulation stages *Wif1* expression appears largely similar in *Xenopus* and zebrafish in the notochord, visceral arches, nasal placodes, swim bladder/lung, otic vesicles, somites (*Xenopus*), lateral line and corneal epithelium (zebrafish) and discrete domains of the brain (Hsieh et al., 1999; Thisse and Thisse, 2005; Yin et al., 2012; Lush and Piotrowski, 2014). In zebrafish, the latter comprise the ventral midbrain and developing dorsal diencephalon (Thisse and Thisse, 2005).

Similarly, in mammals and birds *Wif1* expression initiates relatively late during development and is mainly restricted to the brain, lung, retina, and cartilage (Hsieh et al., 1999; Hunter et al., 2004; Hu et al., 2008; Surmann-Schmitt et al., 2009). In adult mice, *Wif1* expression is retained in the heart and lung and also in the brain and eye, albeit at lower levels (Hsieh et al., 1999). The rather late onset of *Wif1* expression might explain the subtle effects observed in *Wif1* knock out mice, which exhibit accelerated development of radiation-induced osteosarcomas but no recognizable morphological malformations (Kansara et al., 2009). Only in more recent years, mammalian Wif1 was additionally implicated in lung development (Xu et al., 2011), tooth morphogenesis (Lee et al., 2015) and anorectal development (Ng et al., 2014; Figure 1).

The subtle impact on embryonic development and/or maintenance of embryonic structures caused by loss of Wif1 may rather be counterintuitive given its direct interaction with at least six different canonical and non-canonical Wnt ligands and several other proteins (Nakaya et al., 2008; Surmann-Schmitt et al., 2009, 2012). Indeed, forced early ectopic expression of *Wif1* mRNA in the ventral blastomeres of the *Xenopus* embryo causes secondary axes typical for early inhibition of the canonical Wnt signaling cascade (Hsieh et al., 1999). However, Wif1 DNA overexpression leading to ectopic activation at later developmental stages after mid-blastula-transition induces a mild somite phenotype. Furthermore, so far no work has reported a role for Wif1 function in non-canonical Wnt signaling during embryonic development. Thus, Wif1 has the potential to regulate fundamental early processes during axis formation similar to, for instance, Dkk1 (Glinka et al., 1998). However, the onset of its expression mainly after gastrulation implicates that the Wif1/Wnt interaction may only fine-tune the spatial and temporal patterns of Wnt activity (Hsieh et al., 1999). *Wif1* is discretely expressed in cells of tissues, in which Wnt morphogen gradients are at work. For instance, developing neuronal cells in the zebrafish dorsal diencephalon show *Wif1* expression at developmental times, when Wnt3a is active in and around the adjacent mid-diencephalic organizing center to pattern the zebrafish thalamus (Thisse and Thisse, 2005; Mattes et al., 2012). In contrast, cells in the dorsal diencephalon anterior to the thalamus show Wnt activity only later during development (Hüsken et al., 2014). Thus, it is conceivable that particular cells in close vicinity to the Wnt source are protected from premature Wnt signaling – at least for a certain period of time. A Wif1/Wnt feedback regulation for the temporal control of Wnt signaling activity during neurogenesis would be a favorable mechanism to react to dynamic changes within a morphogen gradient.

## Wif1 in Stem Cell Plasticity and as Therapeutic Target in the Eye

Enriched expression of *Wif1* is reported in the cornea of the zebrafish (Thisse and Thisse, 2005), mouse (Davis and Piatigorsky, 2011), monkey (Ding et al., 2008) and human (Nakatsu et al., 2011) eye. Notably, *Wif1* is predominantly expressed in the limbus of the cornea where limbal epithelial stem cells (LESCs) are located (Ding et al., 2008; Nakatsu et al., 2011). LESCs are important for the homeostasis and wound healing capability of the corneal epithelium and hence there is great interest in restoring LESC function in presently untreatable pathologic conditions where corneal healing is impaired (Yazdanpanah et al., 2017). The strong *Wif1* expression in the limbal niche suggests a function in controlling the quiescent state of LESCs under normal physiological conditions (Nakatsu et al., 2011). Removal of Wif1 would allow activation of Wnt signaling associated with the high proliferative behavior of LESCs observed during wound healing and corneal regeneration (Nakatsu et al., 2011). Despite the evolutionarily conserved *Wif1* expression in the cornea and lens (Thisse and Thisse, 2005), surprisingly no studies on the role of zebrafish Wif1 have been reported. However, the combination of transgenes, genetic mutants and *in vivo* time-lapse imaging to visualize dynamic processes under physiological conditions would almost certainly help understanding more about the pathophysiology of LESCs/Wif1/Wnt related diseases. For instance, inducible systems to transiently activate Wif1 in injured wild type or *Wif1* mutant embryos, which carry additional transgenes like *anillin* and signaling reporter to simultaneously highlight cell proliferation (Paolini et al., 2015; Cepero Malo et al., 2017) and Wnt signaling activity (Moro et al., 2012) would be an excellent tool to study Wif1 dependent regeneration processes *in vivo*. The fact that cornea structure, development and maintenance appear comparable between zebrafish and mammals (Pan et al., 2013; Takamiya et al., 2015) should encourage to exploit the zebrafish as *in vivo* model to understand the role of Wif1 in corneal regeneration and homeostasis.

*Wif1* is also expressed in early developing rod photoreceptors, and in the interphotoreceptor matrix of the mature retina (Hsieh et al., 1999; Hunter et al., 2004). Studies from Hsieh et al. suggest that Wif1 plays a role as opposing binding partner of Wnt4, providing a fine-tuning system for the regulation of rod photoreceptor production during development (Hsieh et al., 1999; Hunter et al., 2004). Dysregulated Wnt signaling has been linked to the pathophysiology of neovascular age-related macular degeneration (AMD) as well as diabetic retinopathy (DR) – leading causes of blindness in adults (Chen and Ma, 2017). Intriguingly, increased levels of *Wif1* were found in the aqueous humor of patients with AMD (Park et al., 2014) as well as DR (Kim et al., 2007), suggesting that Wif1 provides an attractive candidate drug target to treat these eye disorders (Figure 1). Furthermore, increasing levels of *Wif1* expression in the vitreous humor correlate with degrees of photoreceptor damage (Park et al., 2014). Alongside being a potential therapeutic target, these studies highlight Wif1 as candidate biomarker of retinal photoreceptor health and disease states.

Other Wif1 functions in the vertebrate eye involve its interaction with members of the Olfactomedin protein family. Within the zebrafish retina, the antagonistic interaction between Wif1 and Olfactomedin 1 in the extracellular space may fine tune retinal ganglion cell axon growth through modulation of Wnt signaling (Nakaya et al., 2008). In the human eye, Wif1 interaction with the Olfactomedin protein family member myocilin appears critical for the regulation of the intraocular pressure (IOP). High IOP in turn is a risk factor for glaucoma and myocilin has been linked to more than 10% of juvenile onset glaucoma cases, which result in the progressive degeneration of the optic nerve (Ortego et al., 1997; Kwon et al., 2009; Menaa et al., 2011).

## Wif1 Regulation During Regeneration

Dynamic changes in *Wif1* expression appear tightly associated to regenerative events in the brain and retina (Gonzalez-Fernandez et al., 2014; Lambert et al., 2016; Yao et al., 2016; Lim et al., 2017). The capability to regenerate lost or damaged neurons in response to injury is a key feature of the fish central nervous system. In the retina, this is achieved through activation and transient asymmetric proliferation of retinal Müller glial cells - the potential stem cells of the retina (Nagashima et al., 2013; Lust et al., 2016). Intriguingly, significant transcriptional down-regulation of *Wif1* and concomitant activation of canonical Wnt signaling was observed in transiently proliferating Müller glial cells (Yao et al., 2016) and also during early stages of zebrafish brain regeneration (Lim et al., 2017; Figure 1). In contrast, damaging the mouse spinal cord appears to induce up-regulation of *Wif1* and other Wnt antagonists (Gonzalez-Fernandez et al., 2014). These data suggest that Wnt signaling after injury might be the key for *de novo* neurogenesis, which is inhibited in the mammalian central nervous system with its limited regenerative capability (Gonzalez-Fernandez et al., 2014 and reviewed in Lambert et al., 2016). The different molecular responses to injury in zebrafish and mammals with respect to Wif1/Wnt signaling and regeneration open exciting possibilities for the development of novel therapeutic approaches to treat central nervous system injuries and possibly also neurodegenerative disorders. Recently, Wif1 has been implicated as potential molecular target of curcumin (Tiwari et al., 2016). Curcumin is a natural polyphenol product derived from the rhizome of the Indian spice turmeric (*Curcuma longa*) that appears to provide neuroprotection in cellular and animal models of neurodegenerative and neurological disorders (Pluta et al., 2015). The capability of curcumin to promote adult neurogenesis, neurite outgrowth and proliferation appears to occur through the interaction with Wif1 and concomitant activation of canonical Wnt signaling (Tiwari et al., 2015).

## Concluding Remarks/Outlook

In summary, the structure and mechanistic underlying Wif1 function is rather well described. Epigenetic silencing of the Wif1 promoter is a common feature, frequently resulting in cancer progression when uncontrolled. Wif1 appears to fine-tune cellular processes, perhaps also temporally controlling them, by dynamically regulating the delicate balance of Wnt signaling via feedback loop activation. Indeed, an increasing number of studies link Wif1/Wnt signaling to different comparably subtle, yet important processes in development and disease, particularly in the eye. Furthermore, Wif1 downregulation and concomitant upregulation of Wnt signaling has been connected to regeneration processes in the zebrafish CNS, while spinal cord lesions in mammals with limited regeneration potential has the opposing effect on *Wif1* expression. Combining the knowledge of the genetic and epigenetic feedback regulation of Wif1 with the regeneration capacities of the zebrafish nervous system has great potential to complement and aid research progress on neural regeneration in mammals.

## Author Contributions

LP and MC had the original idea and outlined the review. All authors contributed to writing the article.

## Conflict of Interest Statement

The authors declare that the research was conducted in the absence of any commercial or financial relationships that could be construed as a potential conflict of interest.
